# Crystal structure of suboptimal viral fragments of Epstein Barr Virus Rta peptide-HLA complex that stimulate CD8 T cell response

**DOI:** 10.1038/s41598-019-53201-6

**Published:** 2019-11-13

**Authors:** Xuelu Huan, Ziyi Zhuo, Ziwei Xiao, Ee Chee Ren

**Affiliations:** 1 Singapore Immunology Network, 8A Biomedical Grove, #03-06 Immunos, Singapore, 138648 Singapore; 2 Department of Microbiology and Immunology, Yong Loo Lin School of Medicine, National University of Singapore, 5 Science Drive 2, Singapore, 119260 Singapore

**Keywords:** X-ray crystallography, Immunology

## Abstract

Peptides presented by Human leukocyte antigen (HLA) class-I molecules are generally 8–10 amino acids in length. However, the predominant pool of peptide fragments generated by proteasomes is less than 8 amino acids in length. Using the Epstein - Barr virus (EBV) Rta-epitope (ATIGTAMYK, residues 134–142) restricted by HLA-A*11:01 which generates a strong immunodominant response, we investigated the minimum length of a viral peptide that can constitute a viral epitope recognition by CD8 T cells. The results showed that Peripheral blood mononuclear cells (PBMCs) from healthy donors can be stimulated by a viral peptide fragment as short as 4-mer (AMYK), together with a 5-mer (ATIGT) to recapitulate the full length EBV Rta epitope. This was confirmed by generating crystals of the tetra-complex (2 peptides, HLA and β2-microglobulin). The solved crystal structure of HLA-A*11:01 in complex with these two short peptides revealed that they can bind in the same orientation similar to parental peptide (9-mer) and the free ends of two short peptides acquires a bulged conformation that is directed towards the T cell receptor. Our data shows that suboptimal length of 4-mer and 5-mer peptides can complement each other to form a stable peptide-MHC (pMHC) complex.

## Introduction

Human leukocyte antigen class-I (HLA-I) molecules are responsible for presenting endogenous peptides mainly generated by proteolytic machinery on the surfaces of cytotoxic CD8^+^ T lymphocytes^1–3^. The peptide binds in a specific groove of HLA-I molecules called as peptide binding groove, which is lined by the conserved sequence of polymorphic residues to stabilize the peptide^4,5^. The peptide is further held in pockets (A - F) present along the groove which determines the specificity of the HLA-I molecules^6^. In particular, peptide binds to pocket-B and pocket-F with the help of anchor residues P2 and PΩ, respectively^6–8^. The ends of the peptide binding groove are closed which is suitable to optimally accommodate peptide repertoire of 8–10 amino acids in length^4,9^. In recent years, some longer peptides (>11-mers) restricted by HLA-I have emerged, where these longer peptides adopt a bulged conformation and are known to comprise 10% of the HLA-I restricted peptide repertoire^10–16^. On the other hand, there are very few instances where shorter peptides (<8-mer) are shown to occupy the peptide binding groove^17–19^. Furthermore, two-thirds of the peptides generated by proteasome are less than 8-mer in length and only less than 15% of the proteasome breakdown products fall within an optimal range of 8–10 mer^20,21^. We have previously determined the occupancy of peptide binding groove for several antigens using synthetic peptides with serial truncation of peptides from both the N- and C-terminal ends^22^. In addition, there are reports demonstrating that non-contiguous peptide presentation by both MHC class I^23^ and MHC class II^24^ bring about an added intricacy in the production of T cell epitopes.

In the present study, Epstein-Barr Virus (EBV) was chosen as a viral model to analyse the immunogenicity of peptide fragments of <8-mer, as EBV is widely circulated in the world population with over 90% of adults being infected^25,26^ and is involved in the manifestation of several types of malignancies^25,27^. We employed a well characterized HLA-A*11:01-EBV restricted peptide ATIGTAMYK derived from BRLF1 protein product Rta (134–142)^28^ to investigate the stability of HLA-A*11:01 molecule in complex with shorter peptides (<8-mer). Rta serves as a transcriptional activator in both B lymphocytes and epithelial cells and is further involved in a switch from latent to the lytic phase^29^. The short peptide fragments derived from the N- and C-terminal regions of ATIGTAMYK (Rta134–142) were synthesized. These shorter peptides, which together make up the complete sequence of the parental peptide, were used alone or in combination to elicit a recall immune response by cytotoxic T lymphocytes. The result shows that short truncated peptides when co-occupying the binding groove complement each other in stabilizing the HLA-I complexes and can evoke antigen specific CD8^+^ T cell response. On the contrary, short truncated peptides alone forms unstable HLA-I complexes and evoke insignificant CD8^+^ T cell response. Furthermore, the crystal structure of HLA-A*11:01 in complex with two short truncated peptides at 1.66 Å demonstrates that they can bind in the same orientation similar to the parent peptide.

## Materials and Methods

### Protein expression and purification

Soluble HLA-A*11:01 (residues 1–274) and full length β2-microglobulin (residues 1–99) with pET30a vectors were expressed in *Escherichia coli* as inclusion bodies using the pET prokaryotic expression system (Novagen Inc.). They were refolded with ATIGTAMYK (9-mer) peptide and were also refolded separately together with two short peptides namely, ATIGT (5-mer) and AMYK (4-mer), and were purified as previously described^30^. Briefly, the refolded peptide-HLA-A*11:01 (pHLA) complexes were dialyzed overnight at 4 °C in 10 mM Tris-HCl (pH 8.0) buffer. The dialyzed pHLA complexes were purified by anion exchange chromatography using HiPrep DEAE 16/10 column (GE Healthcare), and the pHLA complexes were eluted with buffer containing 10 mM Tris-HCl, pH 8.0 and 1 M NaCl. The pHLA complexes were further purified by size exclusion chromatography using HiLoad 16/60 Superdex 75 preparatory-grade column (GE Healthcare) and the pHLA complexes were eluted using buffer 10 mM Tris-HCl, pH 8.0. Finally, the peak corresponding to pHLA complexes were combined and concentrated to 10 mg/ml using Vivaspin centrifugal concentrators (Sartorius).

### HLA-typing, isolation and *in-vitro* culture of human PBMCs

Blood cones from healthy donors were obtained with ethical approval in accordance with Singapore Health Sciences Authority HSA-IRB-201306-5. Donors were recruited with written and informed consent under a Singhealth CIRB Ref: 2017/2512. All methods were performed in accordance with the relevant guidelines and regulations. PBMCs were isolated with Ficoll-Paque PLUS (GE Healthcare) according to manufacturer’s instructions. HLA of donors were identified using sequence based typing as previously described^31^. For 14-day cultures, PBMCs (1 × 10^6^ cells/ml) were incubated with 10 µM peptides (GenScript) and maintained in RPMI 1640 supplemented with 5% human AB serum (Sigma), 100 IU/ml penicillin-streptomycin (Gibco), 2 mM L-glutamine (Sigma), 10 mM HEPES (Sigma) and supplemented with 25 U/ml rIL2 (R&D Systems). Culture medium with rIL2 was replenished every 2–3 days from the fifth day onwards.

### Preparation of peptide-HLA tetramers and cell staining

The peptide-HLA tetramers were prepared as previously described^32^. Briefly, ATIGTAMYK (EBV Rta134–142) peptide was refolded with HLA-A*11:01 heavy chain and β2-microglobulin in refolding buffer for 72 h. Refolded complex was subsequently dialyzed against 10 mM Tris (pH 8.0) at 4 °C overnight. Following purification via anion exchange chromatography using HiPrep DEAE 16/10 column (GE Healthcare) equilibrated with 10 mM Tris-HCl (pH 8.0) and gel filtration with a HiLoad 16/60 Superdex 75 preparatory-grade GF column (GE Healthcare), the pHLA monomeric complexes were biotinylated by recombinant BirA enzymes. Assembly of tetrameric pHLA complexes was carried out by the stepwise addition of streptavidin-phycoerythrin (PE) (Invitrogen) or streptavidin-allophycocyanin (APC) (BioLegend) at a molar ratio of 4:1. Cells from the 14-day cultures were first harvested and then washed with PBS. Subsequently, they were stained with 12 µg/ml PE-conjugated pHLA tetramer for 20 min, and BV421-conjugated anti-human CD8 (BD Biosciences) for 15 min. Cells were washed again with PBS before analysis with LSR II flow cytometer (BD Biosciences). Data analyses were performed using FlowJo (Tree Star Incorporated).

### Thermal stability assay

To evaluate the difference in stability between HLA-A*11:01 bound to 9-mer and dual peptides (5-mer + 4-mer), a thermal shift assay was performed as described previously^33,34^. The thermal stability assay was performed in a real time PCR system LightCycler II 480 (Roche) and the fluorescent dye SYPRO Orange (Molecular Probes) was used to monitor the unfolding of pHLA complexes. Each HLA complexes or control was set in duplicates and the analysis was repeated independently on 2 different batches for the pHLA complexes at a concentration of 20 µM in Tris-HCl buffer (10 mM, pH 8.0), and temperature was increased from 20 °C to 95 °C with continuous ramp rate of 0.04 °C/s with 15 acquisitions/ °C. The fluorescence intensity was measured with excitation at 490 nm and emission at 575 nm. The raw data were normalized and plotted as negative first derivatives of fluorescence change against temperature, whereby, the melting temperature (Tm) is defined as the minima in the negative peak (-dRFU/dT).

### Crystallization

The crystal screening for purified pHLA complexes was initiated using commercial kits (Hampton Research). The pHLA complexes and crystallization solution drops were dispensed in equal volumes (1:1) by employing Phoenix protein crystallization robot (Art Robbins Instruments) in 96 well plates (Art Robbins Instruments) and plates were incubated at 16 °C. After the initial hits were obtained, the crystal drops for pHLA complexes were set using hanging drop vapour diffusion method in 24 well plates (Hampton Research) and plates were incubated at 16 °C. The crystals for HLA-A*11:01-ATIGTAMYK (9-mer) complex were formed in condition containing 0.2 M lithium sulfate, 0.1 M Tris pH 8.5, 30% PEG 4000. For the HLA-A*11:01-ATIGT (5-mer) + AMYK (4-mer) complex, crystals were formed in condition containing 0.2 M sodium malonate pH 5.0, 20% PEG 3350. The pHLA complex crystals were harvested by soaking in a reservoir solution supplemented with 20% glycerol as a cryo-protectant, and stored in liquid nitrogen before use.

### Data collection, processing and structure determination

X-ray diffraction data were collected at the Taiwan Photon Source at National Synchrotron Radiation Research Centre at TPS-05A beamline using the CCD detector MX300-HS. Diffraction data were indexed, integrated and scaled using HKL2000 suite of programs^35^. Both structures were solved by molecular replacement using Phaser^36^ and the previously determined HLA-A*11:01 molecule was used as a search model (Pdb code: 1X7Q)^37^ with peptide and water molecules removed. The model building was performed manually with program COOT^38^ and the refinement was carried out using Refamc5^39^. The quality of the final model was validated using PROCHECK^40^ and figures were prepared with PyMol^41^. The structure refinement statistics is reported in Table 1. The atomic coordinates and structure factors have been deposited in the Protein Data Bank, and the accession code for HLA-A*11:01-ATIGTAMYK is 6JOZ and for HLA-A*11:01-ATIGT + AMYK is 6JP3.

**Table 1:** Data collection and refinement statistics.

Parameter	HLA-A*11:01-ATIGTAMYK^a^ (6JOZ)	HLA-A*11:01-ATIGT + AMYK^a^ (6JP3)
**Data collection**		
Space group	P2_1_2_1_2_1_	P2_1_2_1_2_1_
Cell dimensions		
a, b, c (Å)	51.40, 72.93, 125.70	65.50, 90.53, 91.24
α, β, γ (º)	90, 90, 90	90, 90, 90
Resolution range	30.00–1.35 (1.40–1.35)^b^	50.00–1.66 (1.72–1.66)
Rmeas (%)	8.0 (56.1)	7.0 (85.8)
I/σI	26.2 (3.2)	31.3 (2.4)
CC1/2 (%)	91.8 (72.9)	95.1 (82.4)
Completeness	98.4 (96.5)	99.0 (98.1)
Redundancy	4.4 (2.5)	7.2 (6.7)
**Refinement**		
No. of reflections	96217	60966
Rwork/Rfree	16.0/18.9	16.6/19.4
No. of atoms		
Protein	3100	3146
Peptide	66	66
Glycerol	42	24
Water	518	447
B-factors (Å^2^)		
Protein	17.12	16.74
Peptide	13.36	21.37
Glycerol	24.8	33.50
Water	28.92	30.44
R. m. s. deviations		
Bond lengths (Å)	0.030	0.026
Bond angles (º)	2.492	2.296
Ramachandran plot		
Most favoured (%)	98.11	97.82
Outliers (%)	0	0

^a^Diffraction data from one crystal was merged into a complete dataset.

^b^Numbers in parentheses correspond to the last resolution shell.

r. m. s. deviations: root-mean-square-deviation.

### Accession codes

The atomic coordinates and structure factors for the reported crystal structures have been deposited in the Protein Data Bank under accession codes 6JOZ (HLA-A*11:01-ATIGTAMYK) and 6JP3 (HLA-A*11:01-ATIGT + AMYK).

## Results

### Functional validity of N- and C-terminal truncated peptides

**Figure 1:**
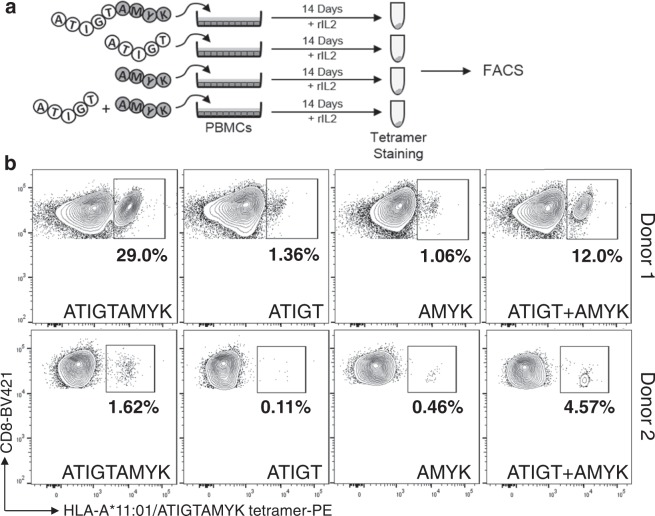
*In-vitro* priming and recall stimulation of human PBMCs using full length and truncated peptides. **(a)** PBMCs were stained with HLA-A*11:01 tetramers formed by using the peptides ATIGTAMYK, ATIGT or AMYK separately, or in combination (ATIGT + AMYK). The plots **(b)** of two different donors represent the frequency of specific CD8+ T cell responses for each tetramer and the values are highlighted in each plot.

Previously, we have shown using short truncated peptides (SSCSSC, 6-mer and SSCPLSK, 7-mer) derived from EBV latent membrane protein-2 epitope, SSCSSCPLSK (LMP2, 340–349) restricted by HLA-A*11:01 to elicit a strong cytotoxic T lymphocytes when presented alone or in combination^22^. Notwithstanding the fact that EBV has the ability to persist and prevail in latent form, however, it will be interesting to investigate how the short peptides originating from the lytic cycle of EBV will behave. Therefore, to evaluate the functionality of the shorter truncated peptides (ATIGT-5-mer and AMYK-4-mer) deduced from the lytic phase of EBV-Rta epitope (ATIGTAMYK, 134–142), PBMCs from two HLA-A*11:01 donors were pulsed with these peptides singly or in combination, and were cultured for 14-days. As shown in Fig. 1a,b, tetramer staining analyses revealed that the truncated peptides were able to stimulate a higher percentage of antigen-specific CD8^+^ T cells in PBMCs when sensitized in combination by two truncated peptides (ATIGT + AMYK, 12.0% for donor 1 and 4.57% for donor 2). The parental 9-mer peptide (ATIGTAMYK) was able to stimulate 29.0% and 1.62% for donor 1 and donor 2 respectively. In comparison, when PBMCs were treated separately with shorter truncated peptides it yielded a lower percentage of antigen-specific CD8^+^ T cells (ATIGT, 1.36% and AMYK, 1.06% for donor 1 and ATIGT, 0.11% and AMYK, 0.46% for donor 2). This provides strong evidence of the presence of specific CD8^+^ T cells from healthy human donor that efficiently recognize the combination of truncated peptides loaded on HLA-A*11:01.

### Crystal structure of HLA-A*11:01-ATIGTAMYK and HLA-A*11:01-ATIGT + AMYK

**Figure 2:**
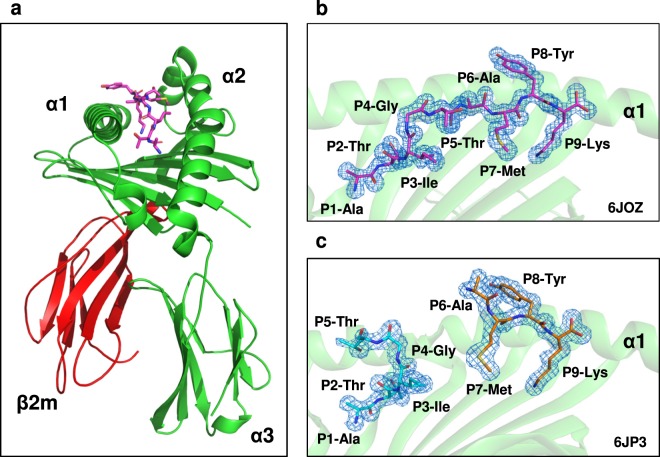
Solved structures and electron density maps of ATIGTAMYK and ATIGT + AMYK peptides bound to HLA-A*11:01. **(a)** The overall structure of HLA-A*11:01 in complex with ATIGTAMYK showing heavy chains (green), β2-microglobulin (red) and peptide (magenta). The panel (b,c) shows electron density map for ATIGTAMYK peptide (magenta) and two short truncated peptides namely ATIGT (cyan) and AMYK (orange) in complex with HLA-A*11:01, respectively. The 2Fo - Fc map is contoured at 1 σ in blue color. The HLA-A*11:01 is represented as a green schematic and for clarity only helix-1 (α1) is shown and the peptides are represented in stick.

To elucidate the structural basis for the two short peptides occupying the same peptide binding groove, the crystal structure of HLA-A*11:01 in complex with two short peptides, namely ATIGT (5-mer) and AMYK (4-mer) was determined at 1.66 Å, and was compared with the structure of HLA-A*11:01 in complex with the 9-mer full-length ATIGTAMYK peptide resolved at 1.35 Å. The overall structure of both pHLA complexes is similar to conventional MHC class I molecules (Fig. 2a)^3,42^. Clear electron densities were observed for the two short peptides ATIGT and AMYK, and also for the full length peptide, ATIGTAMYK within the peptide binding groove of HLA-A*11:01 (Fig. 2b,c).

### Conformation of peptides in the peptide binding groove

**Figure 3:**
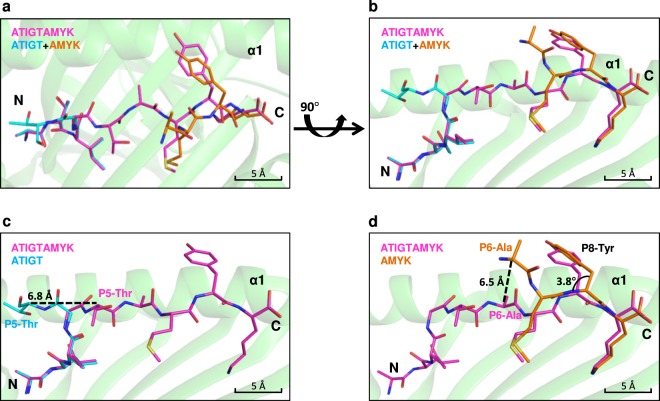
Structural comparison of peptides in the binding groove of HLA-A*11:01. Superposition of peptides showed that short truncated peptides (ATIGT + AMYK) adopted bulged conformation as compared to an extended conformation of parental peptide, ATIGTAMYK. The conformation of the peptides is viewed either from the top **(a)** or via the side **(b)** such that α2 helix is omitted for clarity. The residues, P5-Thr of N-terminal short peptide ATIGT and P6-Ala of C-terminal peptide AMYK have flipped outwards by 6.8 Å **(c)** and 6.5 Å **(d)**, respectively. Furthermore, the ring of P8-Tyr of AMYK (C-terminal, 4-mer) has slanted outward by 3.8° in comparison to P8-Tyr of 9-mer peptide **(d)**. Peptides are shown as sticks, using the same color scheme. The distances between equivalent residues are determined from the respective peptide Cα atoms.

To understand the structural differences between two pHLA complexes, the overall Cα atoms of HLA-A*11:01 in complex with dual peptide (ATIGT + AMYK) was superimposed with HLA-A*11:01-ATIGTAMYK complex and showed an overall r. m. s. d. of 0.38 Å. These small perturbations in two structures can be attributed to conformational flexibility upon peptide binding. However, overlaying only the Cα-atoms of 5-mer peptide (ATIGT) and 4-mer peptide (AMYK) with 9-mer peptide (ATIGTAMYK) showed an r. m. s. d. of 2.05 Å and 0.78 Å, respectively. The 9-mer peptide is seated in a more extended conformation and is held in peptide binding groove in a canonical fashion by primary anchor residues, namely P2-Thr (P2 anchor residue) and P9-Lys (PΩ anchor residue) (Fig. 3a,b). In contrast, the two short peptides ATIGT and AMYK adopted a bulged conformation, whereby, the N-terminal peptide ATIGT contributing P2 anchor residue (P2-Thr) and C-terminal peptide AMYK contributing PΩ anchor residue (P9-Lys) (Fig. 3a,b). The crystal structure of HLA molecules with two short peptides, HLA-A*11:01-ATIGT + AMYK is also compared with HLA molecules with longer peptides (>11-mer), HLA-B*35:01-LPAVVGLSPGEQEY (PDB code: 1XH3), HLA-A*02:01-ALQDAGDSSRKEYFI (PDB code: 4U6X), and HLA-A*02:01-FLNKDLEVDGHFVTM (PDB code: 4U6Y)^12,43^. Several structural studies had previously demonstrated that long peptides (>11-mer) binding to HLA class I alleles displayed high flexibility in the super bulged central region. These results suggest that the free ends of two short peptides acquire a bulged conformation that is directed towards the T cell receptor (Supplementary Fig. 1).

**Figure 4:**
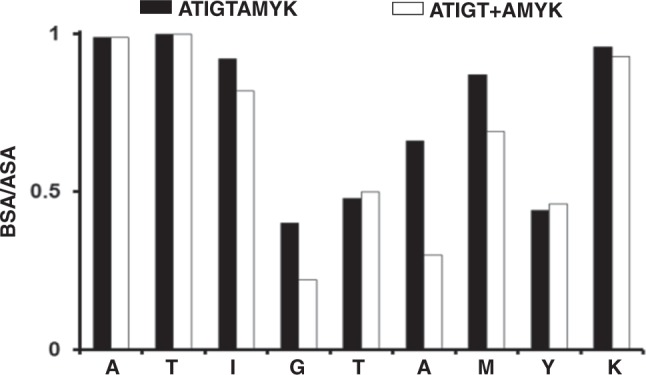
Ratios of the buried surface area (BSA) to the accessible surface area (ASA) of HLA-A*11:01 when bound to ATIGTAMYK (solid bars) or ATIGT + AMYK (open bars) respectively.

Furthermore, the solvent accessible surface area for different peptide residues was calculated with PDBePISA server using a 1.4 Å radius probe (Fig. 4). For HLA-A*11:01-ATIGTAMYK complex, residues P1-Ala (solvent exposure: 0.4 Å^2^), P2-Thr (0.0 Å^2^), P3-Ile (9.3 Å^2^), P7-Met (0.6 Å^2^) and P9-Lys (8.1 Å^2^) show the lowest degree of solvent exposure and are therefore, buried deep in the peptide binding groove. Whereas, residues P4-Gly (49.3 Å^2^), P5-Thr (33.4 Å^2^) and P8-Tyr (59.3 Å^2^) display the highest degree of solvent exposure and are oriented toward the exterior of the binding groove. However, P6-Ala (20.0 Å^2^) exhibit an intermediate level of solvent exposure and is considerably buried in the peptide binding groove.

Similarly, for HLA-A*11:01-ATIGT + AMYK structure, peptide residues P1-Ala (0.4 Å^2^), P2-Thr (0.0 Å^2^), P7-Met (4.3 Å^2^) and P9-Lys (2.9 Å^2^) are minimally exposed to the solvent and resides within the interior of the peptide binding groove. While peptide residues P3-Ile (22.5 Å^2^) and P4-Gly (57.9 Å^2^) display a moderate level of solvent exposure and are markedly buried within the peptide binding groove. In comparison to 9-mer structure, the peptide residues P5-Thr (89.5 Å^2^), P6-Ala (96.5 Å^2^) and P8-Tyr (97.4 Å^2^) of dual peptide structure show maximum amount of solvent exposure and their side chains are pointed outward of the peptide binding groove. Taken together, two short peptides can occupy the same peptide binding groove such that they adopt a bulged conformation similar to HLA molecules with longer peptides (>11-mer)^13^.

### The orientation of nonamer and N- and C-terminal truncated peptides in HLA-A*11:01 groove

The overall orientation of 5-mer peptide is very similar to 9-mer peptide in the peptide binding groove. Nonetheless, P5-Thr of N-terminal peptide ATIGT has flipped outwards by 6.8 Å (Fig. 3c) in comparison to P5-Thr of ATIGTAYMK. Instead of forming a direct hydrogen bond of Gln55 on α2 helix, P5-Thr of N-terminal peptide ATIGT flipped over and is held by forming a direct hydrogen bond with Asn66 on α1 helix. Similarly, overall orientation of the C-terminal peptide AMYK is very similar to 9-mer peptide except that P6-Ala of AMYK has moved outward of the peptide binding groove by 6.5 Å (Fig. 3d) when compared with corresponding P6-Ala of ATIGTAMYK, pointing its side chain outward of the peptide binding groove. Likewise, P7-Met of AMYK has moved to the exterior by 2.5 Å in correlation to the equivalent P7-Met of ATIGTAMYK. While same with P7-Met of ATIGTAMYK, P7-Met of AMYK is stably held by a direct hydrogen bond forming with Gln156 on α2 helix. Furthermore, the head of P8-Tyr of AMYK peptide is slanted at an angle by 3.8° in contrast to the corresponding P8-Tyr of 9-mer peptide, where the head of P8-Tyr in 9-mer is parallel to the peptide binding groove helices (Fig. 3d). As a result, unlike the 9-mer peptide, which seated in a more extended conformation and held in peptide binding groove in a canonical fashion, the free ends of two short peptides acquires a bulged conformation that is directed towards the T cell receptor.

### Stability of the HLA-A*11:01-peptide complexes

**Figure 5:**
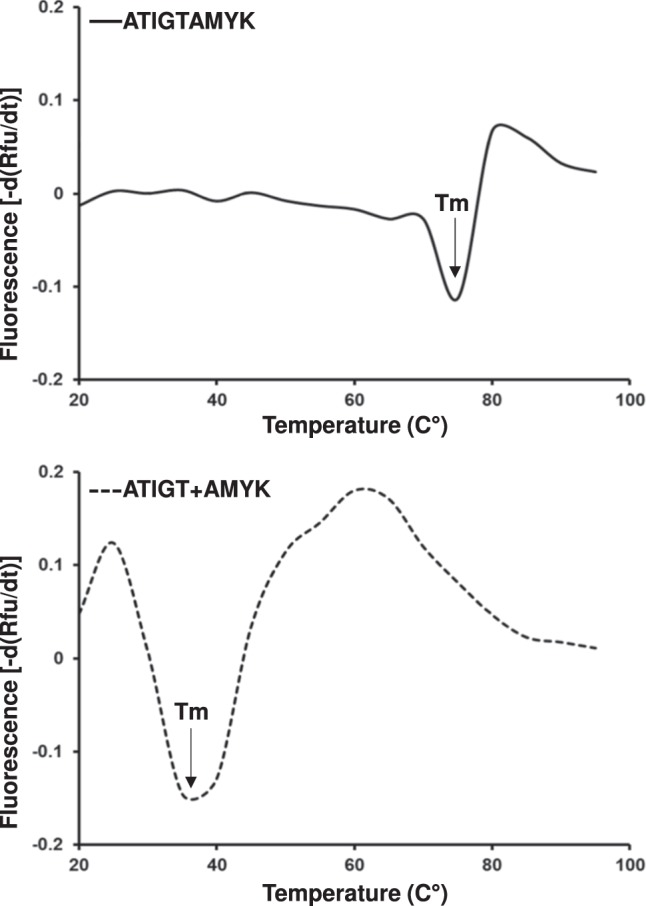
Thermal stability of the HLA-A*11:01-ATIGTAMYK (solid line) and HLA-A*11:01-ATIGT + AMYK (dashed line) was 70 °C and 33 °C, respectively.

To further examine the stability of HLA-A*11:01 in complex with two short peptides (ATIGT + AMYK), a thermal stability assay was performed and compared these values to HLA-A*11:01 bound to a canonical 9-mer peptide (ATIGTAMYK). The pHLA complex unfolding was monitored by using fluorescent dye Sypro orange. The data for each pHLA complex was recorded with two different protein batches, resulting in an average melting temperature (Tm) of 33 °C for HLA-A*11:01-ATIGT + AMYK and 70 °C for HLA-A*11:01-ATIGTAMYK (Fig. 5). The higher Tm-values for 9-mer peptide clearly demonstrate the preference for canonical mode of peptide binding to HLA molecules. However, it is possible for two short peptides to bind to the same peptide binding groove of HLA molecule in a non-canonical manner, but with lesser stability.

### Peptide-binding interactions at P2 and PΩ anchor residues

**Figure 6:**
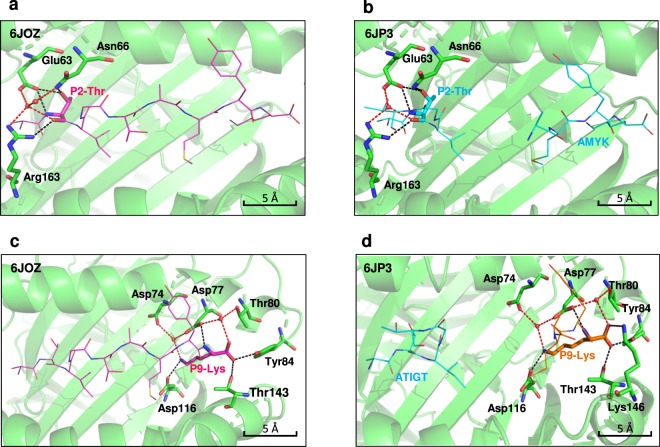
Interactions stabilizing the HLA-A*11:01 peptide binding groove at P2 and PΩ anchor residues. The main chain of HLA-A*11:01 is depicted schematically as cartoon (light green) with selected HLA-I residues shown as sticks. Panel (a) and (b) shows interactions between P2 anchor residues (P2-Thr) of ATIGTAMYK and N-terminal truncated peptide ATIGT, respectively with HLA-A*11:01. Similarly, panel (c) and (d) details interactions between PΩ anchor residue (P9-Lys) in both the structures, respectively. The hydrogen bonding in indicated as black colored dotted line, whereas, interactions mediated via water molecules are indicated as red colored dotted line. Peptides are displayed as sticks and lines following the same color scheme and only P2 or PΩ anchor residue is labelled. The water molecules are shown in red spheres.

The various peptides in the study, namely, ATIGTAMYK (9-mer), ATIGT (5-mer) and AMYK (4-mer) are held in the peptide binding groove by forming stable interactions with HLA-A*11:01 molecule essentially by primary anchor residues P2-Thr and P9-Lys, either directly or mediated via water molecules. Briefly, the pocket B of peptide binding groove of HLA-A*11:01 where primary anchor P2 is seated is lined with polymorphic residues which are suitable to hold small aliphatic amino acids like valine, leucine, serine as well as threonine^44^. The side chain of P2-Thr of 9-mer forms direct hydrogen bond with Glu63 and Asn66, as well as through its main chain amino group it is directly hydrogen bonded to Glu63. Additionally, the main chain carbonyl group of P2-Thr is stably held by forming a direct hydrogen bond with Arg163. Furthermore, P2-Thr interacts with Glu63, Asn66 and Arg163 via framework of two water molecules (Fig. 6a). On the other hand, P2 anchor residue threonine of N-terminal peptide ATIGT in dual peptide structure is stabilised by similar bonding pattern as P2-Thr of 9-mer structure, except that it does not make any water mediated interaction with Asn66 (Fig. 6b).

The F-pocket of peptide binding groove of HLA-A*11:01 is characterized by negatively charged backdrop made up of Asp74, Asp77 and Asp116 residues, and which is suitable to accommodate positively charged residues, lysine or arginine. This is evident from a salt-bridge contact between the side chain Nζ-ammonium group of P9-Lys and Asp116. The side chain of P9-Lys also bonds with Asp74 and Asp77 via water molecules. Additionally, main chain amino group of P9-Lys makes a direct hydrogen bond with Asp77 and carboxylate of P9-Lys forms direct hydrogen bond with Tyr84 and Thr143, and also interacts with Asp77 and Thr80 via water molecules (Fig. 6c). In comparison with the 9-mer structure, the C-terminal peptide AMYK in a dual peptide structure is held stably in a similar manner in the F pocket by corresponding PΩ anchor residue P9-Lys except that carboxylate makes an additional direct hydrogen bond with Lys146 (Fig. 6d).

## Discussion

Classically, HLA-I molecules present peptides with 8–10 amino acids in length with their N and C termini bound in the A and F-pockets, respectively^3,45^. The cell’s proteasome machinery is responsible for the generation of the bulk of peptide source for HLA-I molecules^46^ but only 15% of proteasome breakdown product falls within an optimal range of 8–10 residues^20^. Another 10% of the proteasomal products are longer than 11 amino acids, and in the recent years the evidence of longer peptides (>11-mer) occupying the peptide binding groove is emerging^13^. Moreover, the majority two-thirds of all peptides produced by proteasome are shorter than 8 residues^20,21^ and fate of these short peptides is not very well documented in terms of their contribution to HLA-I molecule peptidome. The present study has provided functional and structural evidence to display that HLA-I molecules can bind short peptides (<8-mer) and present them to specific cytotoxic T lymphocytes (CTLs).

In this study we chose a very well characterized T cell epitope, ATIGTAMYK (9-mer) originated from the EBV-Rta protein (134–142) to examine the peptides that are competent in evoking T cell responses^32^. Rta (immediate early transactivator) is the product of BRLF1, and is associated with the reactivation of EBV from latency and in impelling the EBV during lytic phase^29^. During the lytic phase, Rta evokes the cellular immune response in EBV carriers. The data presented here demonstrates that synthetic shorter peptides (ATIGT 5-mer; AMYK 4-mer) derived by truncation of parental peptide (ATIGTAMYK 9-mer) when presented alone stimulated a negligible percentage of T cell responses from PBMCs of healthy HLA-A*11:01 positive donors previously exposed to EBV. When these two shorter truncated peptides are presented in combination they elicit a higher CD8^+^ T cell recall response. Furthermore, the cellular immune response by canonical 9-mer parental peptide (ATIGTAMYK) is relatively lesser when compared with shorter truncated peptides presented in combination. Taken together, shorter peptides can complement each other when presented in tandem and may contribute in stabilizing the peptide-HLA complex. This notion is supported by a recent report suggesting that progressively truncated short peptides from immunogenic EBV antigen SSCSSCPLSK can still bind to HLA-I molecule and elicit CTLs despite having lower affinities^22^. Likewise, short peptides derived from L^d^ peptide ligands, tum^−^ and p2Ca are also shown to bind MHC class-I molecule as complementary pairs and stimulate specific CTLs^18^.

The crystal structure of HLA-A*11:01 in complex with two short complementary peptides (ATIGT 5-mer and AMYK 4-mer) indicates that short synthetic peptide pairs can bind in similar orientation as the original longer parental peptide (ATIGTAMYK). Interestingly, these short complementary peptide pair adopt a bulged conformation in the centre similar to longer peptides (>11-mer)^13^ whereby, the residues in the centre are highly solvent exposed, and could serve as a potential contact point for T cell receptors (TCR). Accordingly, it will be of great interest to know how T-cells can recognize short complementary peptide pairs. The TCR could likely bind in a diagonal orientation as generally accepted^45,47^ or it will predominantly focus on peptides^48^ and accommodate the dual peptide fragments in a different way.

Our analysis of short synthetic peptides co-occupying the same HLA-I binding groove is of considerable interest in devising the strategy for the development of vaccines and immunotherapy. It demonstrates the flexibility of HLA-I groove to accommodate short peptides of different sizes and as short as 4-mer while maintaining the specificity of T cell recognition. Furthermore, there is a possibility of one of the peptide pair originating from self-antigens, and in combination with non-self-antigens it can give rise to neo-epitopes with far wider implications on autoimmune disorders. In addition, these short peptide pairs may originate from the same protein or different proteins, or may be non-contiguous as in the case of spliced peptides^23^ or hybrid peptides^24^. In summary, we demonstrated that HLA-A*11:01 can bind two short synthetic peptides derived from EBV epitope and this pHLA complex can evoke recall response of specific CTLs *in vitro*. This combine-and-complement pair of peptides can contribute towards the existing pool of HLA-I immunopeptidome giving new insights about peptide presentation, and potentially augmenting the possibilities to generate new vaccine design and immunotherapy.

## Supplementary information

Supplementary Figures 1

Supplementary Tables 1
