# Supplementary material for: Crystal structure of suboptimal viral fragments of Epstein Barr Virus Rta peptide-HLA complex that stimulate CD8 T cell response
Source: Sci Rep. 2019 Nov 13;9:16660. doi: 10.1038/s41598-019-53201-6 (PMC6853878; doi:10.1038/s41598-019-53201-6)
Supplement: Supplementary file 1 — Supplementary Figures 1 [file 41598_2019_53201_MOESM1_ESM.pdf]

Crystal structure of suboptimal viral fragments of Epstein Barr Virus Rta peptide-HLA complex that stimulate CD8 T cell response

Xuelu Huan[1], Ziyi Zhuo[1], Ziwei Xiao[1], Ee Chee Ren*[1, 2]

[1]Singapore Immunology Network, 8A Biomedical Grove, #03-06 Immunos, Singapore 138648
[2]Department of Microbiology and Immunology, Yong Loo Lin School of Medicine, National University of Singapore, 5 Science Drive 2, Singapore 119260

Correspondence: Dr EC Ren, Singapore Immunology Network, 8A-Biomedical Grove, #03-06 Immunos, Singapore 138648.
Email: ren_ee_chee@immunol.a-star.edu.sg
Phone: (65)64070004
Fax: (65)64642056

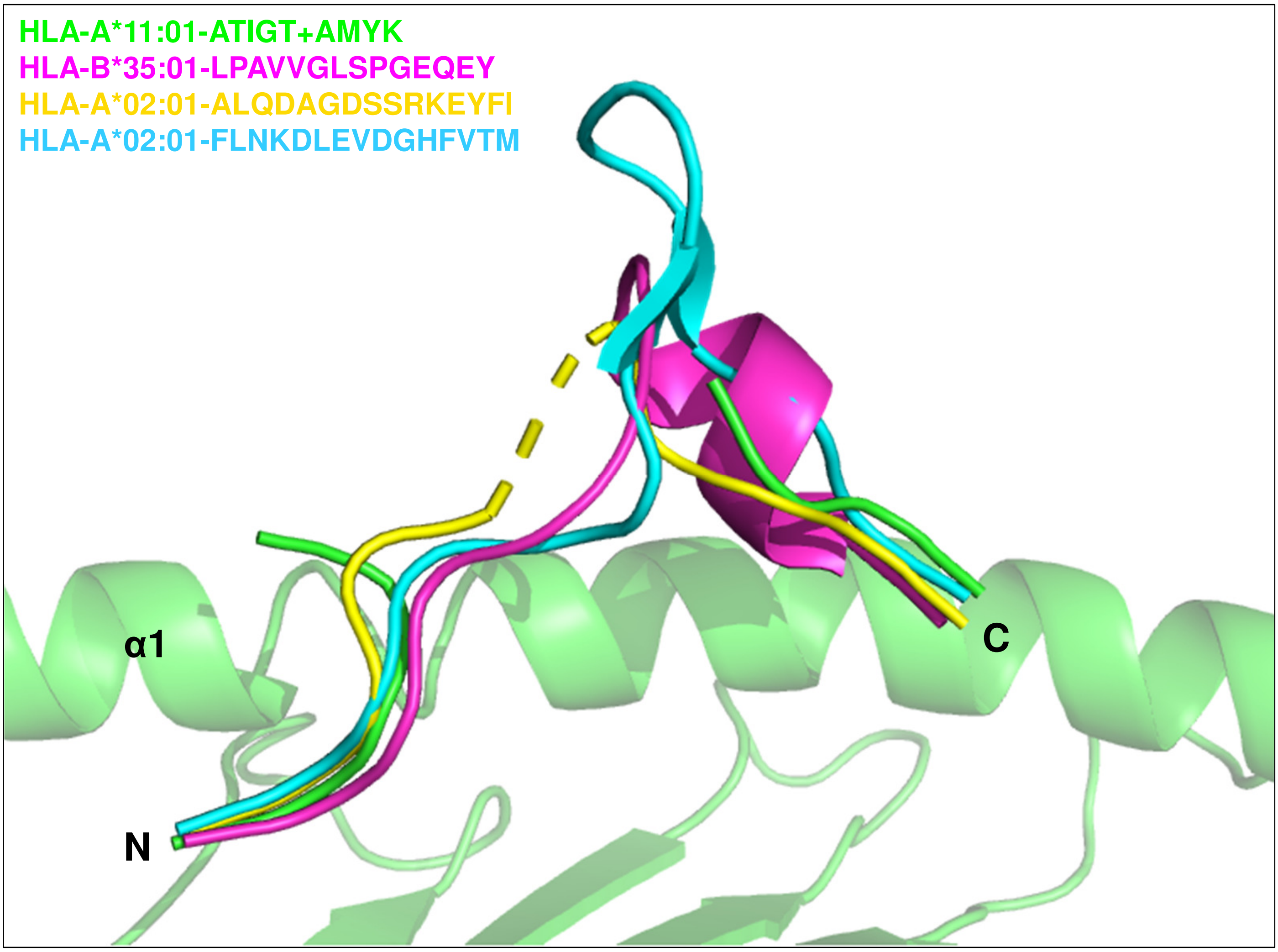

Supplementary Figure 1. The crystal structure of HLA-A*11:01-ATIGT+AMYK (green; PDB code: 6JP3) is compared with HLA molecules with longer peptides (>11-mer), HLA-B*35:01-LPAVVGLSPGEQEY (magenta; PDB code: 1XH3), HLA-A*02:01-ALQDAGDSSRKEYFI (yellow; PDB code: 4U6X), and HLA-A*02:01-FLNKDLEVDGHFVTM (cyan; PDB code: 4U6Y).

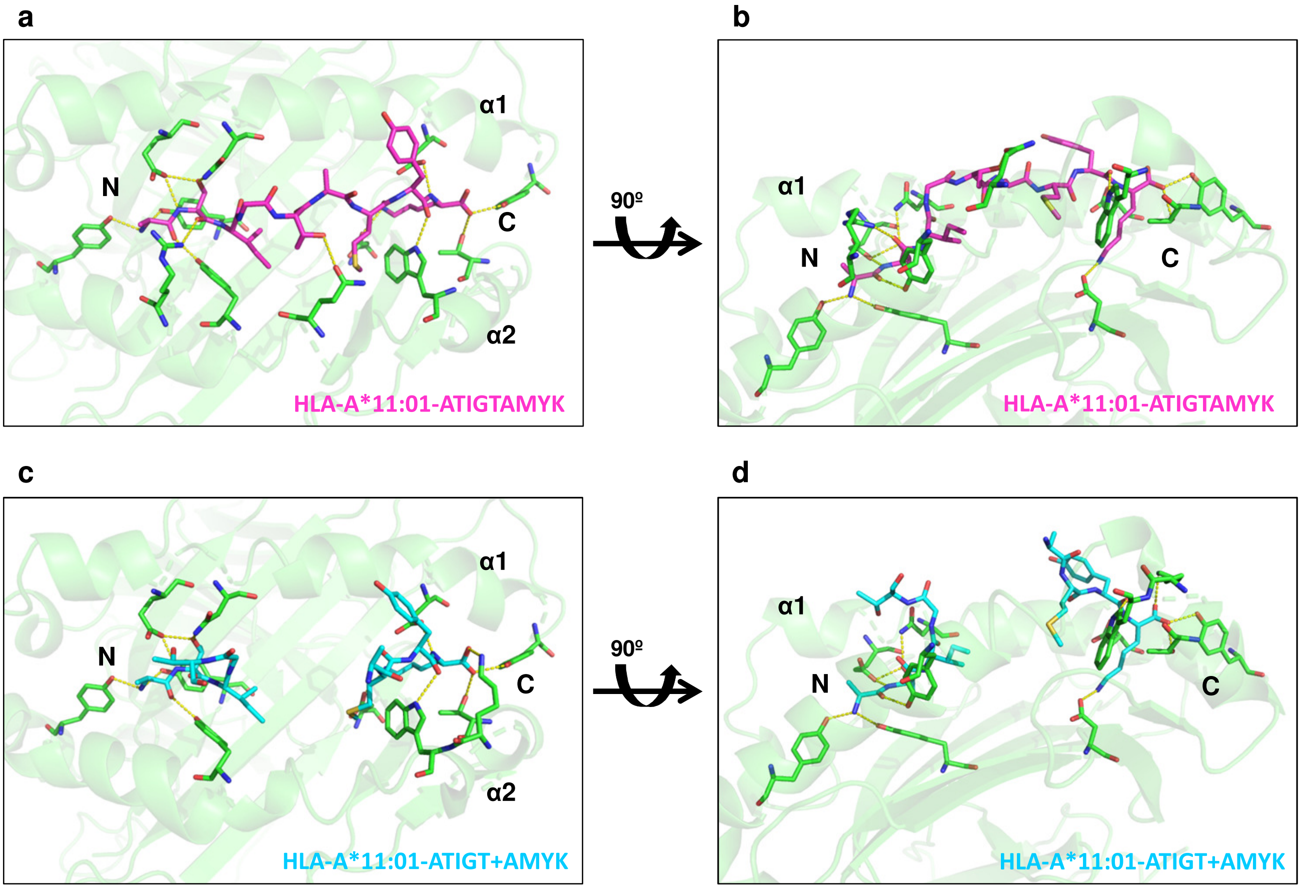

Supplementary Figure 2. The detailed interaction maps of HLA-A*11:01-ATIGTAMYK and HLA-A*11:01-ATIGT+AMYK, viewed from the top (**a**) and via the side (**b**) for HLA-A*11:01-ATIGTAMYK, and viewed from the top (**c**) and via the side (**d**) for HLA-A*11:01-ATIGT+AMYK.
