# Supplementary material for: Crystal structure of suboptimal viral fragments of Epstein Barr Virus Rta peptide-HLA complex that stimulate CD8 T cell response
Source: Sci Rep. 2019 Nov 13;9:16660. doi: 10.1038/s41598-019-53201-6 (PMC6853878; doi:10.1038/s41598-019-53201-6)
Supplement: Supplementary file 2 — Supplementary Tables 1 [file 41598_2019_53201_MOESM2_ESM.pdf]

Crystal structure of suboptimal viral fragments of Epstein Barr Virus Rta peptide-HLA complex that stimulate CD8 T cell response

Xuelu Huan[1], Ziyi Zhuo[1], Ziwei Xiao[1], Ee Chee Ren*[1, 2]

[1]Singapore Immunology Network, 8A Biomedical Grove, #03-06 Immunos, Singapore 138648

[2]Department of Microbiology and Immunology, Yong Loo Lin School of Medicine, National University of Singapore, 5 Science Drive 2, Singapore 119260

Correspondence: Dr EC Ren, Singapore Immunology Network, 8A-Biomedical Grove, #03-06 Immunos, Singapore 138648.

Email: ren_ee_chee@immunol.a-star.edu.sg

Phone: (65)64070004

Fax: (65)64642056

Supplementary Table 1

Hydrogen bond and Van der Waals interaction between ATIGTAMYK peptide and HLA-A*11:01 residues.

Peptide		Binding Partner[a)]		vdW Contact Residues[b)]
Residue	Atom	Residue	Atom	
P1-Ala	N<br><br>O	Tyr7<br>Tyr171<br>Tyr159	OH<br>OH<br>OH	Met5, Tyr7, Glu63, Tyr159, Trp167, Tyr171
P2-Thr	N<br><br>OG1<br><br>O	Tyr7<br>Glu63<br>Glu63<br>Asn66<br>Arg163	OH<br>OE1<br>OE1<br>ND2<br>NH2	Tyr7, Tyr9, Met45, Glu63, Asn66, Tyr99, Tyr159, Arg163
P3-Ile	N<br>O	Tyr99<br>Asn66	OH<br>ND2	Asn66, Tyr99, Gln156, Tyr159
P4-Gly				Asn66
P5-Thr	OG1	Gln155	OE1	Gln155
P6-Ala				Gln70, Thr73
P7-Met	SD	Gln156	NE2	Thr73, Asp77, Trp133, Trp147, Ala152, Gln156,
P8-Tyr	O	Trp147	NE1	Thr73, Val76, Asp77, Lys146, Trp147
P9-Lys	N<br>NZ<br>O	Asp77<br>Asp116<br>Tyr84<br>Thr143	OD1<br>OD2<br>OH<br>OG1	Asp77, Thr80, Leu81, Tyr84, Ile95, Asp116, Thr143, Lys146, Trp147

a) The cut off distance for hydrogen bond interactions is 3.5 Å.

b) The cut off distance for van der Waals (vdW) contact is 4.0 Å.

a), b) The distances between various interactions was measured using CONTACT from ccp4i suite and COOT.

Supplementary Table 2

Hydrogen bond and Van der Waals interaction between N-terminal short peptide ATIGT (1-5) and HLA-A*11:01 residues.

**Peptide**		**Binding Partner**[a)]		**vdW Contact Residues**[b)]
**Residue**	**Atom**	**Residue**	**Atom**	
P1-Ala	N<br><br>O	Tyr7<br>Tyr171<br>Tyr159	OH<br>OH<br>OH	Met5, Tyr7, Glu63, Tyr159, Arg163, Trp167, Tyr171
P2-Thr	N<br><br>OG1<br><br>O 	Tyr7<br>Glu63<br>Glu63<br>Asn66<br>Asn66<br>Arg163	OH<br>OE1<br>OE1<br>ND2<br>ND2<br>NH2	Tyr7, Tyr9, Met45, Glu63, Asn66, Tyr99, Tyr159, Arg163
P3-Ile	N<br>O	Tyr99<br>Asn66	OH<br>ND2	Asn66, Tyr99, Gln156, Tyr159
P4-Gly				Asn66
P5-Thr	OG1	Gln62<br>Asn66	NE2<br>ND2	Gln62, Asn66, Arg163

[a)] The cut off distance for hydrogen bond interactions is 3.5 Å.

[b)] The cut off distance for van der Waals (vdW) contact is 4.0 Å.

[a), b)] The distances between various interactions was measured using CONTACT from ccp4i suite and COOT.

Supplementary Table 3

Hydrogen bond and Van der Waals interaction between T-terminal short peptide AMYK (6-9) and HLA-A*11:01 residues.

Peptide		Binding Partner[a)]		vdW Contact Residues[b)]
Residue	Atom	Residue	Atom	
P6-Ala				
P7-Met	SD	Gln156	NE2	Thr73, Trp147, Ala152, Gln155, Gln156,
P8-Tyr	O	Lys146<br>Trp147	NZ<br>NE1	Thr73, Val76, Asp77, Lys146, Trp147
P9-Lys	N<br>NZ<br>OT1<br><br>OT2	Asp77<br>Asp116<br>Tyr84<br>Lys146<br>Tyr84<br>Thr143	OD1<br>OD2<br>OH<br>NZ<br>OH<br>OG1	Asp77, Thr80, Leu81, Tyr84, Ile95, Asp116, Thr143, Lys146, Trp147

[a)] The cut off distance for hydrogen bond interactions is 3.5 Å.

[b)] The cut off distance for van der Waals (vdW) contact is 4.0 Å.

[a), b)] The distances between various interactions was measured using CONTACT from ccp4i suite and COOT.
